# Clinical Pharmacology of Midazolam in Neonates and Children: Effect of Disease—A Review

**DOI:** 10.1155/2014/309342

**Published:** 2014-02-18

**Authors:** Gian Maria Pacifici

**Affiliations:** Section of Pharmacology, Department of Translational Research and New Technologies in Medicine and Surgery, Medical School, University of Pisa, 56126 Pisa, Italy

## Abstract

Midazolam is a benzodiazepine with rapid onset of action and short duration of effect. In healthy neonates the half-life (*t*_1/2_) and the clearance (Cl) are 3.3-fold longer and 3.7-fold smaller, respectively, than in adults. The volume of distribution (Vd) is 1.1 L/kg both in neonates and adults. Midazolam is hydroxylated by CYP3A4 and CYP3A5; the activities of these enzymes surge in the liver in the first weeks of life and thus the metabolic rate of midazolam is lower in neonates than in adults. Midazolam acts as a sedative, as an antiepileptic, for those infants who are refractory to standard antiepileptic therapy, and as an anaesthetic. Information of midazolam as an anaesthetic in infants are very little. Midazolam is usually administered intravenously; when minimal sedation is required, intranasal administration of midazolam is employed. Disease affects the pharmacokinetics of midazolam in neonates; multiple organ failure reduces the Cl of midazolam and mechanical ventilation prolongs the *t*_1/2_ of this drug. ECMO therapy increases *t*_1/2_, Cl, and Vd of midazolam several times. The adverse effects of midazolam in neonates are scarce: pain, tenderness, and thrombophlebitis may occur. Respiratory depression and hypotension appear in a limited percentage of infants following intravenous infusion of midazolam. In conclusion, midazolam is a safe and effective drug which is employed as a sedative, as antiepileptic agent, for infants who are refractory to standard antiepileptic therapy, and as an anaesthetic.

## 1. Introduction

Midazolam is a short-acting benzodiazepine with rapid onset of action. It has anxiolytic, muscle relaxant, and anticonvulsant activity, now most widely used to generate anterograde amnesia and to stop prolonged seizures in children [1]. Midazolam is one of the most widely used sedatives in the “neonatal intensive care unit” [2]. The sedative and anticonvulsant properties of midazolam are related to GABA accumulation and occupation of benzodiazepine receptors [3]. Antianxiety properties are related to increasing the glycine inhibitory neurotransmitter [3].

Midazolam exerts most of its effects by interacting with inhibitory neurotransmitter receptors directly activated by GABA. GABA_A_ receptors are responsible for most inhibitory neurotransmission in the central nervous system. Benzodiazepines act at GABA_A_ receptors by binding directly to a specific site that is distinct from that of GABA binding [4]. Midazolam, which was approved for clinical use in 1976, acts as a sedative-hypnotic, is used in the treatment of refractory seizures, and induces anaesthesia [3].

In adults, the half-life (*t*_1/2_) of midazolam is 1.9 hours, which is 22-fold shorter than that of diazepam [5]. Bioavailability of midazolam is about 50% when absorbed directly through either oral or nasal mucosa [6]. Midazolam is mainly eliminated by hydroxylation to form 1-hydroxymidazolam by CYP3A4 and CYP3A5 enzymes. 1-Hydroxymidazolam has sedative effect [4]. Finally, this metabolite is glucuronidated before excretion into urine [7]. Hepatic CYP3A4 activity appears in the liver during the first weeks of life [8, 9]; it is thus lower in neonate than adult liver, resulting in reduced midazolam clearance (Cl) in neonates [6, 10]. CYP3A5 expression has been detected in only 10% to 30% of neonate liver samples tested [11]. Elimination *t*_1/2_ of midazolam is shorter in neonates; it is about from 4 to 6 hours. It is quite variable and may be up to 22 hours in premature infants [3]. CYP3A4 and CYP3A5 activities reach adult levels between 3 and 12 months of postnatal age [9]. Midazolam is highly bound to plasma protein; in adults, the bound percentage is 98 [5].

The literature on the effects and fate of midazolam in neonates is scarce. Most of the published information deals with the sedative effect, with the treatment of refractory seizures to standard therapies, with the metabolism, with the pharmacokinetics, and with the adverse effects of midazolam. The information on the use of midazolam as an anesthetic agent in newborn infants is lacking. Mellon et al. [12] reviewed the anaesthetic agents in newborn animals. Information on the anaesthetic activity of midazolam has been reported in Sprague-Dawley rats [13] and in mice C57BL6 [14]; both animals were 7 days old. No data on the anaesthesia by midazolam in infants are available.

Infants may experience moderate to severe pain in the “neonatal intensive care unit” and the use of a sedative to make their life comfortable when undergoing painful procedures is useful [15]. Midazolam was found to be more effective than placebo as a sedative in neonates [16] and its concentrations ranging from 100 to 400 ng/mL are sufficient for sedation [17]. Jacqz-Aigrain et al. [16], Anand et al. [18], and Arya and Ramji [19] used midazolam for sedation of neonates undergoing mechanical ventilation and midazolam has resulted to be a safe and effective sedation.

Benzodiazepines provide effective control of patient agitation and these drugs are useful to comfort neonates during stressing procedures. Midazolam represents a convenient choice among the sedatives because of its fast onset of action and rapid termination of effect and is thus frequently used in the “neonatal intensive care units.”

Up to now, little has been done to make comfortable the life of neonates who undergo stress and pain. Lowrie et al. [20] created a “pediatric sedation unit” to uniform and monitor the therapy for sedation and/or analgesia for children undergoing invasive and noninvasive procedures. Infants admitted to the “pediatric sedation unit” were assessed medically for risk factors during sedation. Many of the pediatric patients admitted to this unit were neonates. Pharmacological treatment helps neonates to tolerate pain procedures or diagnostic studies requiring prolonged periods of immobility. The majority of children needing sedation received midazolam or propofol [20].

Ng et al. [21] reviewed the literature on the intravenous midazolam infusion for sedation of infants in the “neonatal intensive care unit.” Three trials were included in the study and using different sedation scales each study showed a higher sedation level in the midazolam group compared to the placebo. However, Ng et al. [21] conclude that there are insufficient data to promote the use of intravenous midazolam infusion as a sedative for neonates undergoing intensive care [22].

Sedation may be performed with opioids (fentanyl, morphine, and diamorphine) or with the sedative-hypnotic midazolam in ventilated neonates. Endotracheal intubation and mechanical ventilation are major components of routine intensive care for very low birthweight newborn infants and sick fullterm newborn infants [22]. Sedation is widely used in neonates, although there is limited clinical evidence for the efficacy and safety of the drugs used or the methods of administering them [22].

A useful action of midazolam is the management of neonatal seizures refractory to conventional treatments. Hu et al. [23] reported that a continuous intravenous infusion of midazolam, ranging from 1 to 15 *μ*g/kg per min, with an average of 4 *μ*g/kg per min, terminated the seizures in 32 infants whose seizures could not be controlled by diazepam, phenytoin, or phenobarbital. Castro Conte et al. [24] found that midazolam (0.15 mg/kg intravenous bolus, followed by a continuous infusion of 1 *μ*g/kg per min, increasing by 0.5 to 1 *μ*g/kg every 2 min, until a maximum of 18 *μ*g/kg per min) controlled the seizures in 13 neonates refractory to the treatment with phenobarbital and phenytoin.

Troponin T is a prognostic indicator of postoperative recovery. Midazolam, propofol, or sevoflurane had similar efficacy in the production of troponin T in infants who underwent cardiac surgery [25].

The information on the effects of midazolam and on the fate of this drug in neonates has been published in different journals during the last thirty years and it is scattered. The aims of this article are (1) to gather together and (2) to review the published data on (a) the metabolism and (b) the pharmacokinetics of midazolam in neonates, (c) the therapeutic effects, and finally (d) the adverse effects of midazolam in neonates. The main objective of this work is (e) to provide neonatologists with a tool that embraces all aspects of the clinical pharmacology of midazolam in neonates. Little is known about the effects and the fate of the drugs administered to neonates. This review was written to help the neonatologists, hoping that they will find some useful information and thus some advantages for their work, from reading the present paper.

## 2. Bibliographic Search

The bibliographic search was performed electronically using PubMed and EMBASE databases as search engines; September 2013 was the cutoff point. The following key words: “midazolam neonate,” “midazolam mechanism of action,” “midazolam therapy neonate,” “midazolam pharmacokinetics neonate,” “midazolam metabolism neonate,” “CYP3A4 midazolam neonate,” “CYP3A5 midazolam neonate,” “oral administration midazolam neonate,” “intranasal administration of midazolam neonates,” “midazolam seizures,” “midazolam anaesthesia neonate,” and “midazolam adverse effects neonate,” were used. The bibliography of each article was read carefully, and the selected articles were examined. In addition, the books NEOFAX: a Manual Used in the Neonatal Care, by Young and Mangum [3], and the Neonatal Formulary [1] were consulted. The findings of the bibliographic search gave rise to 101 original articles, 13 review articles, and 6 book chapters. The publication years of this matter ranged from 1979 to 2013.

## 3. Results

### 3.1. Doses of Midazolam in Neonates

For sedation, Young and Neofax [3] suggest giving intravenously a 50 to 150 *μ*g/kg of midazolam over at least 5 min. Midazolam may also be administered intramuscularly. Repeat as required, usually Q2 to 4 hours. Dosage requirements are decreased by concurrent use of narcotics. For continuous intravenous infusion, give a 10 to 60 *μ*g/kg midazolam per hour. Dosage of this drug may need to be increased after several days of therapy because of development of tolerance and/or increase of Cl. For Intranasal administration, give a 200 to 300 *μ*g/kg midazolam per dose using 5 mg/mL injectable form. For sublingual administration, give a 0.2 mg/kg per dose of midazolam using 5 mg/mL injectable form mixed with small amounts of flavoured syrup. For Oral administration, give 250 *μ*g per dose. For Anticonvulsant therapy; give intravenously a loading dose of 150 *μ*g/kg over at least 5 min followed by a maintenance infusion dose of 60 to 400 *μ*g/kg per hour (1 to 7 *μ*g/kg per min).

### 3.2. Metabolism of Midazolam in Neonates

Development has a profound effect on the expression of phase I enzymes such as the cytochromes P450 (CYPs). Distinct patterns of isoform specific development CYP expression have been observed postnatally. Although the CYP content in the fetal liver equals about 30 to 60% of adult values [35], isoforms have a specific ontogeny and preclude the generalisation of a simple single developmental pattern for overall CYP activities, necessitating isoenzymes specific assessment [36, 37].

In adults, midazolam is metabolized rapidly [7]; the recovery of unchanged midazolam in urine of adult subjects is 1% [5] suggesting that metabolism is the main route of midazolam elimination in man. Midazolam is hydroxylated to form 1-hydroxymidazolam by hepatic CYP3A4 [3, 38–41] and by CYP3A5 [38, 40–43]. The oxidative metabolism of midazolam is an aliphatic hydroxylation [38]. A significant correlation was observed between the fraction of 1-hydroxymidazolam excreted into urine and the postconceptional age [10].

Plasma Cl of midazolam is widely used as an in vivo surrogate measurement of CYP3A4 and CYP3A5 activities [44, 45]. Cl of midazolam is lower in preterm infants than in older children and adults [2]. The percent recovery of midazolam into the urine is higher in infants (0.44%) than in adults (0.14%), confirming that the metabolic rate of midazolam is reduced in infants. CYP3A4 activity surges in the liver during the first week of life [8, 9]. Therefore, the expression of CYP3A4 in the neonatal liver is lower than that in the adult. CYP3A5 expression shows large interindividual variability and has been detected in only 10% to 30% of neonate liver samples tested [11].

Ince et al. [46] developed a novel maturation function for midazolam Cl based on studies in premature neonates, infants, toddlers, children, adolescents, and adults. This model provides a quantitative insight in the development pattern of in vivo CYP3A activities across the pediatric range, including premature neonates. The largest change in Cl of midazolam, which is metabolised by CYP3A4/5 in vivo and in vitro, appears in the first week of life [40]. Ince et al. [46] studied the interactions of hepatic CYP3A activity using midazolam as an in vivo probe. The effect of ontogeny on intestinal CYP3A activity is still unknown; intestinal CYP3A is of considerable importance. The knowledge of the maturation of CYP3A mediated first pass elimination of midazolam is important to understand the contribution of the intestine to the liver to the first-pass elimination of midazolam.

The ontogeny of midazolam glucuronidation was studied in 22 preterm infants [10]. Midazolam (100 *μ*g/kg) was administered intravenously (*n* = 15) or orally (*n* = 7). After intravenous administration, the median percentage of the dose excreted into the urine, over 6-hour intervals, was 0.44% (midazolam), 0.04% (1-hydroxymidazolam), and 1.57% (1-hydroxymidazolam glucuronide). After oral administration, the median percentage of the dose excreted into the urine was 0.11% (midazolam), 0.02% (1-hydroxymidazolam), and 1.69% (1-hydroxymidazolam glucuronide). The glucuronidation of 1-hydroxymidazolam is immature in preterm infants less than 2 weeks of postnatal age.

### 3.3. Pharmacokinetics of Midazolam in Neonates and Children

The Cl of midazolam was measured in 24 preterm infants and the mean was 1.8 (mL/kg per min) [26]. In children with age > 2 years, the Cl of midazolam was 9.6 ± 3.5 mL/kg per min (range: 5.8 to 13.6 mL/kg/min) [17, 47] and 6.6 mL/kg per min in adults [5]. The lower Cl of midazolam observed in neonates is due to the lower expression of CYP3A4 and CYP3A5 in neonatal liver [9, 11, 48, 49].

The published literature on the pharmacokinetics of midazolam in neonates is scarce and the available information is heterogeneous because the work was done in healthy neonates [26], in critically ill infants [2, 6, 27–29], or in infants undergoing extracorporeal membrane oxygenation (ECMO) [30, 31].

The information on the pharmacokinetics of midazolam summarised in this review is organised on the neonate health status, and it is reported in (a) healthy neonates, (b) in critically ill neonates (c), and in infants undergoing extracorporeal membrane oxygenation (ECMO) and finally (d) the population pharmacokinetics of midazolam are reviewed in neonates. The pharmacokinetic parameters of midazolam are summarised in Table 1.

### 3.4. Pharmacokinetics of Midazolam in Healthy Neonates

The pharmacokinetics of intravenous midazolam (100 *μ*g/kg) were studied in 24 healthy preterm infants, with a gestational age between 26 and 34 weeks [26]. The maximal concentration of midazolam in plasma (Cmax) and the time to reach Cmax (Tmax) were 108 ng/mL (range: 48.8 to 217 ng/mL) and 0.5 hours (range: 0.5 to 4.0 hours), respectively. For midazolam, *t*_1/2_ was 6.3 hours (range: 2.6 to 17.7 hours), Cl was 1.8 mL/kg per min (range 0.7 to 6.7 mL/kg per min), Vd was 1.1 L/kg (range: 0.4 to 4.2 L/kg), and AUC_0–∞_ was 971 ng·h/mL (range: 248 to 2353 ng·h/mL). In the text, these values are referred to as the “normal values” or the “normal estimates.”

No significant relationships were detected between age (gestational, postnatal, or postconceptional) and midazolam Cl, Vd, and *t*_1/2_. For 1-hydroxymidazolam, Cmax was 8.2 ng/mL (range: <0.5 to 68.2 ng/mL) and Tmax was 6 hours (range: 1 to 12 hours). The median 1-hydroxymidazolam AUC_0–*t*_ to midazolam AUC_0–*t*_ ratio was low (0.09) and showed a large interindividual variation (range: <0.001 to 1). There were no relationships between 1-hydroxymidazolam pharmacokinetic parameters and midazolam Cmax, AUC, and *t*_1/2_. No 4-hydroxymidazolam could be detected.

Anderson and Larsson [50] described a maturation model for midazolam Cl in neonates and children. Cl maturation, standardized to a 70 kg person, was described using the Hill equation. Mature Cl was 523 (CV32%) mL/min per 70 kg. The maturation *t*_1/2_ was 73.6 weeks. Predicted Cl changes with age, based on this model, were in close agreement with physiologically based pharmacokinetic models. Previously published pharmacokinetic parameters can be used to develop maturation models that address gaps in current knowledge regarding the influence of age on a drug's disposition. If midazolam sedation target concentration of 100 ng/mL, similar to that given to adults, is assumed, we might anticipate steady-state infusion rates of 14 *μ*g/kg per hour in neonates, 50 *μ*g/kg per hour in a 1-year-old infant, 60 *μ*g/kg per hour in a 5-year-old infant, and 50 *μ*g/kg per hour in a 12-year-old child. Age-related pharmacodynamic differences that will affect dose and the impact of active metabolites on response have not yet been quantified.

### 3.5. Pharmacokinetics of Midazolam in Critically Ill Neonates and Children

Disease reduces the midazolam elimination rate in neonates. In critically ill neonates, undergoing mechanical ventilation, *t*_1/2_ was 9.8 hours, after a 200 *μ*g/kg bolus of midazolam, and 12 hours, after a constant infusion of 60 *μ*g/kg per hour of midazolam [28]. In normal values *t*_1/2_ is 6.3 hours. These estimates are 155% and 190%, respectively, of the normal values [26].

A 15.1-year-old girl, suffering from renal failure, with malignant hypertension and with a creatinine concentration of 691 *μ*mol/L, received an intravenous bolus of 100 *μ*g/kg midazolam. *t*_1/2_, Cl, and Vd of midazolam of this girl were 6.3-fold longer, 2.6-fold greater, and 14.5-fold larger, respectively, [34] than the normal values [26]. The patient's mean of 1-hydroxymidazolam plus 1-hydroxymidazolam glucuronide was more than 3 times above the population value [34].

A 1.1-year-old boy, suffering from pneumonia, received the CYP3A4 inhibitor erythromycin and the CYP3A4 substrate fentanyl. He had a *t*_1/2_ and a Cl of midazolam of 3.8-fold longer and 1.8-fold greater, respectively, [34] than the normal values [26].

Vet et al. [27] determined the effects of inflammation and disease severity on midazolam pharmacokinetics and pharmacodynamics in 21 critically ill children aged between 2 days and 17 years. Midazolam continuous infusion was 90 *μ*g/kg per hour (range: 50 to 270 *μ*g/kg per hour). The pharmacokinetic parameters of midazolam are summarised in Table 1. Eleven severely critically ill 2.5-year-old patients (range: 0.1 to 9.0 years) needed midazolam for sedation. They were suffering from congenital heart disease, upper airway infection, pneumonia, postcardiac surgery and pulmonary hypertension, and other diseases. Midazolam Cl was significantly lower in these children (median = 0.14 L/kg per hour) than in 10 patients with less severe disease; their median Cl was 0.28 L/kg per (*P* = 0.035). A significant negative correlation was found between disease severity and midazolam Cl corrected for body weight (*r* = −0.49; *P* = 0.02). These results suggest that increased disease severity is associated with reduced midazolam Cl. Likely, these results reflect a reduction of CYP3A activity.

Alternative explanations could be the altered protein binding, midazolam being highly bound to plasma proteins (98% in adults, [5]), or, less likely, reduced blood flow, because midazolam has an intermediate extraction rate [51].

The pharmacokinetics of midazolam was studied in 15 preterm infants undergoing mechanical ventilation for respiratory distress syndrome [28]. Midazolam was administered as an intravenous continuous infusion of 60 *μ*g/kg per hour; the infusion lasted for 60.0 ± 23.3 hours. In these patients, *t*_1/2_ estimate was double the normal value [26]. Hypotension, associated with a slight reduction of heart rate, was observed in 4 cases after midazolam administration [28]. Mean systolic and diastolic blood pressure fell from 59/40 to 35/25 mm Hg. In 3 infants, the hypotension occurred immediately after the administration of midazolam bolus dose. In these three infants, the midazolam plasma concentrations were 0.17, 0.58, and 1.24 *μ*g/mL; in two cases, the patients received fentanyl before midazolam. The metabolic ratio of midazolam to 1-hydroxymidazolam, was 16.1 ± 21.2 (*n* = 11) at 48 hours after the administration. Various drugs were coadministered with midazolam during the study; they were fentanyl (*n* = 7), antibiotics, in most cases betalactams and aminoglycosides (*n* = 13), albumin (*n* = 5), and vasopressin drugs (*n* = 3). Jacqz-Aigrain et al. [28] conclude that the elimination of midazolam was markedly delayed in premature infants with respiratory distress syndrome undergoing mechanical ventilation. However, these authors did not take into consideration the possible effects of the drugs coadministered with midazolam. In particular, fentanyl, a substrate for CYP3A4 [52], can inhibit the metabolism of midazolam increasing its *t*_1/2_.

Ten critically ill preterm infants, with a postnatal age of 2 to 5 days, received a single intravenous bolus dose of 200 *μ*g/kg of midazolam [29]. Midazolam was well tolerated during and after the intravenous administration. There was no clinical evidence of any adverse effects after midazolam administration, and the heart rate and the arterial blood pressure remained unchanged during the study. *t*_1/2_, Cl, and Vd were 6.5 ± 2.3 hours, 2.0 ± 1.2 mL/kg per min, and 0.9 ± 0.3 L/kg, respectively. The kinetic parameters in these infants were similar to those of normal values [26].

Ince et al. [43] performed a population pharmacokinetic modelling using a joint dataset of 3 studies. Fifty-four children, aged between 1 month and 17 years, who received intravenous midazolam (bolus and/or continuous infusion) for sedation were enrolled in the study. The parameter estimates from the final model were used for simulation and to predict the concentrations from the following midazolam schedules (intravenous bolus of 100 *μ*g/kg followed by an intravenous infusion of either 50 *μ*g/kg per hour, for postoperative monitoring, or 100 *μ*g/kg per hour for conscious sedation). In children, a large variability in the pharmacokinetic parameters of midazolam was observed. Midazolam is a substrate for CYP3A4 and CYP3A5 and the expression of these enzymes considerably varies in neonates [6, 10, 11].

The wide variability of midazolam pharmacokinetic parameters cannot be explained by age-related changes alone. Ince et al. [43] studied these age-related changes in relation to other covariates to explain the variability in the pharmacokinetics of midazolam. The following covariates were tested for all parameters: bodyweight, postnatal age, specific subpopulation, and mechanical ventilation (yes/no). Midazolam, 1-hydroxymidazolam, and 1-hydroxymidazolam glucuronide concentrations were considered to determine the pharmacokinetics of midazolam and metabolites using NONMEN 6.2. A SimCYP pediatric simulator was used for simulation. A reduction of 93% for CYP3A4/5 (midazolam to 1-hydroxymidazolam) and 86% for uridine diphosphate glucuronosyltransferase (1-hydroxymidazolam to 1-hydroxymidazolam glucuronide) mediated Cl was found in pediatric intensive care patients compared with the other 2 patient groups. Ince et al. [43] did not find a significant influence of age or body weight on CYP3A4/5 mediated total Cl. For uridine diphosphate glucuronosyltransferase mediated Cl, body weight explained 41.5% of the variability. From infancy to adolescence, critical illness seems to be the major determinant of midazolam Cl, which may result from reduced CYP3A4/5 activity due to inflammation. This may have important implications for dosing of midazolam and other CYP3A drug substrates in critically ill children.

A new dosing schedule was designed, aiming for midazolam concentrations between 45 and 64 ng/mL for postoperative monitoring after procedural sedation [53] and between 205 and 307 ng/mL for conscious sedation in “neonatal intensive care unit” patients [54]. These concentrations correspond with previously reported adequate sedation levels of the respective population groups and are used for indicative purposes only.

### 3.6. Pharmacokinetics of Midazolam in Neonates Undergoing Extracorporeal Membrane Oxygenation (ECMO)

Extracorporeal membrane oxygenation (ECMO) is a form of prolonged cardiopulmonary bypass used to support patients with life-threatening respiratory or cardiac failure [55]. In neonates, ECMO is used for a variety of indications, including sepsis and pulmonary disease such as meconium aspiration syndrome, persistent pulmonary hypertension, or congenital hernia [55].

ECMO yields variability and lack of predictability in drug behaviour. The most common mechanisms by which ECMO affects pharmacokinetics are sequestration of the drug in the circuit and the increase of drug Cl and Vd [55, 56]. The pharmacokinetic parameters of drugs undergoing ECMO vary during this therapy. Thus, Table 1 gives two values for each pharmacokinetic parameter, an estimate was obtained at the start, and another one was obtained at the end of ECMO therapy.

Adequate sedation of neonates receiving ECMO is essential to allay the physical, emotional, and psychologic distress experienced during intensive care. Midazolam, a short-acting benzodiazepine derivative with rapid onset of action, is the sedative of choice and is preferred over other benzodiazepines because of its water solubility and the perceived rapid elimination [30].

Twenty neonates with gestational age of 39.5 ± 1.9 weeks, postnatal age of 3.8 days, and body weight of 3,400 ± 600 g were enrolled in the study [30]. The infants were randomized into two groups: group 1 (*n* = 10) received midazolam extracorporeally (into the circuit), via a pigtail catheter, whereas group 2 (*n* = 10) received the drug via a central or peripheral venous catheter. Midazolam was administered as a continuous infusion at a rate between 50 and 250 *μ*g/kg per hour, initiated as soon as cannulation was achieved and extracorporeal blood flow was established. The pharmacokinetic parameters obtained by Mulla et al. [30] are summarised in Table 1.

The mean metabolic ratio was of 1-hydroxymidazolam to midazolam 0.17 (range: 0.03 to 0.9) and appeared to be significantly higher than that previously reported in non-ECMO infants (0.09), and it was similar to those observed in older children and adults (range: 0.13 to 0.25 [57, 58]).

DeBerry et al. [59] determined the general practice guideline used for pain and anxiolytic pharmacotherapy of pediatric patients at ECMO centers. Fentanyl was the most commonly used drug for pain medication and continuous infusion. However, midazolam was considered to be the most effective agent used.

Ahsman et al. [31] studied 20 neonates undergoing ECMO. The median gestational age, postnatal age, and bodyweight were 40.4 weeks, 0.79 days, and 3,000 g, respectively. Midazolam (200 *μ*g/kg) was administered by a venoarterial catheter as a bolus infusion before cannulation. The median ECMO duration was 124 hours (range: 70 to 275 hours). When discomfort occurred, midazolam was started as a continuous infusion of 100 *μ*g/kg per hour. The midazolam dose was incrementally adjusted (with steps of 100 *μ*g/kg per hour) on the basis of the required level of sedation, which was objectified with the validated COMFORT behaviour scale. Once the patient was fully sedated, the midazolam infusion was interrupted until COMFORT scores indicated that additional sedation was necessary.

Midazolam Cl and Vd rapidly increased in 5 days of ECMO therapy. The kinetic parameters of midazolam are summarised in Table 1. In contrast, the Vd estimates for 1-hydroxymidazolam and 1-hydroxymidazolam glucuronide remained constant at 10.2 L and 1.2 L, respectively.

To quickly reach stable serum concentrations of 400 ng/mL, the optimal dose regimen would be a continuous infusion of 300 *μ*g/kg per hour for the first 6 hours, after which, the infusion rate should be reduced to 150 *μ*g/kg per hour. After 5 days, the infusion rate should be increased to 200 *μ*g/kg per hour to compensate for the continual increasing of midazolam Cl and 1-hydroxymidazolam Cl. 1-Hydroxymidazolam glucuronide accumulated during ECMO, providing an increased proportion of the overall effect, up to 34% after 7 days of ECMO therapy. Large unexplained interindividual variability warrants careful titration of sedation effects. Ahsman et al. [31] did not find any effect of serum creatinine on pharmacokinetic parameters. The use of creatinine as a marker of renal function in newborn infants is in dispute [60, 61].

### 3.7. Population Pharmacokinetics of Midazolam in Neonates and Children

In literature there are three studies of midazolam population pharmacokinetics [32–34]. One hundred and eighty-seven neonates, requiring intravenous sedation for mechanical ventilation, were enrolled in the study [32]. Their gestational and postnatal ages ranged from 26 to 42 weeks of amenorrhea and from 0 to 9 days, respectively. The birthweight ranged from 700 to 5,200 g. Midazolam was administered intravenously as a continuous infusion of 69 ± 93 *μ*g/kg per hour (range: 11 to 603 *μ*g/kg per hour) to 109 infants; the infusion lasted for 6 to 171 hours (62 ± 31 hours). A bolus dose of 210 ± 239 *μ*g/kg (range: 32 to 1,600 *μ*g/kg) was administered to 22 infants, and a combination of both was administered to 56 neonates. The mean midazolam doses required for critically ill neonates are lower than those required for wealthy neonates. Mean *t*_1/2_ was 9.9 hours, Cl was 1.2 ± 0.2 mL/min/kg, and Vd was 1.0 ± 0.2 L/kg [32]. There was a large interindividual variability in the Cl (CV, 17%) and Vd (CV, 20%). Compared with the population estimate, Cl was 1.6 times higher in neonates with a gestational age of more than 39 weeks and was 0.7-fold lower in neonates receiving inotropic support. The postnatal age had no apparent effect on midazolam pharmacokinetics. The estimates of the population pharmacokinetic parameters and their interindividual coefficients of variation (CV) without covariates were Cl = 0.107 L/kg (CV, 83%), Vc (central volume) = 0.788 L (CV, 92%), and Vp (peripheral volume) = 0.969 L (CV, 163%). Birthweight was clearly correlated with Cl and Vc and, to a lesser extent, with Vp. The authors did not give the level of significance of these correlations. The gestational age correlated with the birthweight (*r* = 0.89; *P* = 0.0001). The 531 measured plasma concentrations of midazolam ranged from 0 to 7,100 ng/mL; 105 samples were above 1,000 ng/mL. Midazolam was not detected in 19 samples. Aminoglycosides, aminopenicillins, and third generation cephalosporins increased the plasma midazolam concentrations for 73.8%, 72.2%, and 48.7%, respectively. Aminoglycoside treatment was associated with a 50% decrease in midazolam Cl.

Lee et al. [33] studied the population pharmacokinetics in 60 premature infants undergoing mechanical ventilation. Their mean gestational age was 27 weeks (range: 24 to 31 weeks), their postnatal age was 4.5 days (range: 2 to 15 days), and their body weight was 965 g (range: 523 to 1,470 g). A rapid intravenous bolus of 100 *μ*g/kg was administered every 4 to 6 hours. Average parameter values for infants with birthweight of 1,000 g were as follows. Systemic Cl was 0.783 mL/min (CV = 83%) and Vd of central comportment was 473 mL (CV = 70%). *t*_1/2_ was not given. For infants with body weight more than 1,000 g, Cl was 1.24 mL/min (CV = 78%) and Vd was 823 mL (CV = 43%). The average population Cl in boys (0.642 mL/min) was less than in girls (0.808 mL/min). The level of significance of this difference was not given. The population mean Cl of overall patients was 0.938 mL/kg per min. The average Vd of overall infants was 1.15 L/kg, which agreed closely with the Vd of 1.0 L/kg in term and preterm infants [32].

The population kinetics of midazolam was studied in eighteen pediatric patients, aged from 2 days to 17 years [34]. Midazolam was administered as an intravenous bolus of 100 *μ*g/mL followed by a continuous infusion ranging from 50 to 400 *μ*g/kg per hour, for 3.8 hours to 25 days for conscious sedation. *t*_1/2_, Cl, and Vd of midazolam were 5.5 ± 3.5 hours, 5.0 ± 3.9 mL/kg per min, and 1.7 ± 1.1 L/kg, respectively. Half of the patients had an age less than 6 months [34]. Mean Cl rate is lower than that reported in patients with age greater than 2 years (mean ± SD = 9.6 ± 3.5 mL/kg per min [61, 62]).

The Cl reported by de Wildt et al. [34] is comparable to that obtained in another study performed with pediatric intensive care patients, aged between 26 days and 5 years (mean ± SD = 5.8 ± 3.8 mL/kg per min [17]), when studied during the first 24 to 48 hours of midazolam infusion. These data support an age-related increase of midazolam Cl, consequent to an age-related surge in CYP3A4 and CYP3A5 activities [49].

1-Hydroxymidazolam to midazolam ratio (mean ± SD = 0.21 ± 0.20, [34]) is in agreement with the ratio obtained in pediatric patients who received midazolam after cardiac surgery (mean ± SEM = 0.25 ± 0.03 [59]). In contrast, the ratio obtained by Wildt et al. [34] was higher than that in newborn infants <2 weeks of age during continuous infusion of midazolam (0.06 ± 0.05 [28]).

### 3.8. Pharmacodynamics of Midazolam in Neonates

Very little is known about the pharmacodynamics of midazolam in infants and only two articles are available in literature [54, 63]. Swart et al. [63] stated that from the clinical studies it is clear that there is a large variability in response to a given concentration of midazolam. Vet et al. [27] did not find a correlation between inflammation severity and COMFORT score. Trouiller et al. [64] and Arbor et al. [65] said that the optimal measure to monitor the pharmacodynamic endpoint still needs to be determined. Dosing regimens in children are based upon rather empirical extrapolations from the dosing regimens in adults. Several authors have developed scaling methods to predict midazolam Cl as a function of age, taking ontogeny into account [66–68]. From these data it can be concluded that body weight increases with age from approximately 0.06 L/kg per hour in preterm neonates to 0.6 L/kg per hour in children 5 years of age before decreasing to 0.4 L/kg per hour in adult patients.

Twenty-one infants, aged from 2 days to 17 years, received a continuous infusion of midazolam from 50 to 400 *μ*g/kg per hour, for a period of 3.8 hours to 25 days [54]. A possible pharmacokinetic-pharmacodynamic relationship for midazolam in pediatric intensive care patients and the determination of how adequate sedation could be reached using the COMFORT scale was studied. Sedation levels were determined using the COMFORT scale as well as the plasma concentrations of midazolam and metabolites. An evident pharmacokinetic-pharmacodynamic relationship was not found. In 20 of the 21 patients studied, midazolam dosing could be effectively titrated to the desired level of sedation, assessed by the COMFORT scale. Based on these findings, there is no relationship between pharmacodynamic parameters and pharmacodynamic outcome. De Wildt et al. [54] recommend that midazolam dosing should be titrated according to the desired clinical effect combination with a validated assessment instrument such as the COMFORT scale. The COMFORT scale rates 6 behavioural and 2 physiologic dimensions of distress, each scored on a subscale from 1 to 5. Thus, a maximum score is considered reflective of oversedation, between 17 and 26 as effective/optimal sedation, as proposed by Marx et al. [69]. As reflected by the Pediatric Risk of Mortality (PRISM) score, disease severity among our patients varied considerably. Seven patients required analgesia (morphine, codeine, fentanyl, and acetaminophen) during the midazolam infusion. No significant relationship between sedation level category and drug or metabolite concentrations was detected. The ranges of midazolam study (50 to 400 *μ*g/kg per hour) were in agreement with other data from studies in pediatric intensive care patients [70, 71].

### 3.9. Sedation of Neonates and Children by Midazolam

Neonates can perceive pain when mechanically ventilated: this fact has been demonstrated by the physiological, biochemical, and behavioural changes indicative of pain and stress in newborn infants during mechanical ventilation [72]. Pain induces agitation in the newborn, and the newborn's agitation may contribute to asynchrony between the newborn and the ventilator. To sedate neonates with midazolam this drug is usually given intravenously or by intranasal administration. The oral administration of midazolam in neonates is little used and, in literature, only two articles on oral midazolam in neonates are available [2, 6]. No data of sublingual administration of midazolam in neonates was found in literature.

D. A. Rosen and K. R. Rosen [73] wrote a review on the sedation with midazolam in conscious pediatric patients. These authors stated that the advantages of midazolam include quick onset and short duration of action and hemodynamic stability which may be associated with improved patient acceptance. The comparison with diazepam showed that midazolam has a more controlled sedation, with a quicker recovery time. Midazolam yields a shorter recovery and less vomiting than diazepam in children who underwent surgery. The safety and tolerability profile of midazolam in pediatric patients is comparable or superior to that observed in adults. Patients who are haemodynamically unstable, as well as preterm and term infants, are at greater risk of hypotension while receiving sedation. Midazolam provides an important additional option for providing intravenous conscious sedation in the pediatric population.

The American Academy of Pediatrics Committee on Drugs has issued guidelines categorizing pharmacological intervention into three levels: conscious sedation, deep sedation, and general anaesthesia [74]. Conscious sedation refers to a state of depressed consciousness that “allows protective reflexes to be maintained” and “permits appropriate responses by the patient to physical stimuli or verbal commands.” Deep sedation is defined in part as “partial or complete loss of protective reflexes” and lack of purposeful response to physical stimulation. Deep sedation requires the presence of personnel skilled in airway management and cardiopulmonary resuscitation [20].

Intravenous infusion of benzodiazepines is important to assist ventilation and other potentially distressing procedures. Combined with intermittent or continuous infusion with opioids, the benzodiazepines provide smooth control of anxiety, pain, and agitation [75]. The major adverse effects of long-term benzodiazepine infusion are withdrawal symptoms and delayed awakening. Intravenous midazolam represents an important choice among drugs used for sedation in the “neonatal intensive care unit” for short-term use in infants undergoing assisted ventilation and generally rapid awakening when the drug is discontinued [76]. Midazolam and lorazepam are the most frequently employed benzodiazepines in the “neonatal intensive care unit.” These two drugs are distinguished from the other benzodiazepines by their relatively higher water solubility, rapid onset, and short duration of action [47]. These pharmacologic properties of midazolam facilitate regulation of the level of sedation. Midazolam is usually given by continuous infusion, following a loading dose.

Treluyer et al. [77] determined the minimal effective dose of intravenous midazolam required for appropriate sedation in 95% of patients, 1 hour after drug administration. Twenty-three newborn infants, hospitalized in intensive care unit, were enrolled in the study. Their gestational age was >33 weeks and midazolam was administered as an intravenous bolus at a dose from 75 to 200 *μ*g/kg followed by a maintenance dose ranging from 37.5 to 100 *μ*g/kg per hour over 47 hours. The sedation procedure was classified as a success if all the following clinical criteria were met: no agitation, no grimacing, and no crying facial expression before as well as during tracheal suctioning. The estimated success probability, updated after each patient, was <95% for all dose levels and led to allocation of the maximum loading dose (200 *μ*g/kg). Based on the 23 patients studied, the final estimated probability of success was 76.9% (95% credibility interval was 56.6 to 91.4%) for the 200 *μ*g/kg loading dose. The posterior success probability for the six doses 75, 100, 125, 150, 175, and 200 *μ*g/kg corresponded to 27.7%, 32.7%, 38.1%, 47.3%, 52.5%, and 76.9%, respectively. Failures were recorded in 5 (25%) of 20 patients receiving a 200 *μ*g/kg midazolam loading dose. No significant effect of sedation on pulse oximetry, oxygenation index, or triggering of ventilator breathing was evident after the onset of midazolam administration. No significant adverse effects were observed. Pneumothorax was reported in two children who had received a 200 *μ*g/kg initial loading dose and were classified as “success” for sedation. A decrease of more than 30% for systolic, diastolic, and mean blood pressure was observed during the study inclusion in none, two (8.7%), and one (4.3%) children, respectively (all received the 200 *μ*g/kg loading dose of midazolam).

Forty-six infants undergoing mechanical ventilation for respiratory distress syndrome were randomly assigned to receive midazolam (*n* = 24) or placebo (*n* = 22) as a continuous infusion [16]. Jacqz-Aigrain et al. [16] performed a prospective placebo controlled study of the effects of midazolam on hemodynamic variables and sedation as judged by a five-item behaviour score. Doses of midazolam were calculated to obtain plasma concentrations between 200 and 1000 ng/mL within 24 hours of starting treatment and to maintain these values throughout the study. In the treatment group, infants of 33-week gestation or more received a continuous infusion of 60 *μ*g/kg per hour of midazolam. Infants below 33-week gestation received the same continuous infusion of 60 *μ*g/kg per hour during the first 24 hours of treatment, followed by a continuous infusion of 30 *μ*g/kg per hour of midazolam. The assigned treatment lasted for 5 days, but sedation with midazolam could be continued if prescribed by the attending neurologist. Midazolam gave a significantly better sedative effect than placebo (*P* < 0.05). Heart rate and blood pressure were reduced by treatment but remained within the normal range for gestational age and there was no effect on ventilatory indices. Hemodynamic instability requiring albumin and/or vasoactive drugs occurred in 14 infants, 8 of whom were in the midazolam group and 6 infants were in the placebo group. Intraventricular haemorrhage occurred in 8 infants in the midazolam group and 6 infants in the placebo group. Blood samples were obtained from 23 of 24 infants. Mean midazolam concentrations were 634 ± 234 ng/mL, 628 ± 327 ng/mL, and 543 ± 327 ng/mL at 24 and 48 hours and at the end of treatment, respectively. Substantial variability in plasma concentrations of midazolam was seen, but none of the infants had values below 200 ng/mL and only 2 infants (with a gestational age below 32 weeks) had a plasma concentration of midazolam above 1000 ng/mL. There was no correlation between sedation score and corresponding midazolam concentration. No midazolam-related adverse effects were noted. Continuous infusion of midazolam at doses adapted to gestational age induces effective sedation in newborn infants on mechanical ventilation, with positive effects on hemodynamic variables. Midazolam was given over only a few days and the limited effects on heart rate and blood pressure that were reported should not encourage long-term administration.

Midazolam was administered as a continuous infusion at a dosage of 50 to 400 *μ*g/kg per hour, to 24 infants, partly in combination with fentanyl (0.5 to 2.5 *μ*g/kg per hour) for analgesia and sedation [17]. The mean duration of midazolam infusion was 11.6 days (range: 38 hours to 40 days). The efficiency of sedation in correlation with the midazolam concentration was evaluated by a clinical sedation score. Serum midazolam concentrations from 100 to 400 ng/mL were sufficient for sedation. Dosage had to be increased during therapy according to an increased midazolam Cl. The evaluation of sedation score showed that sedation of artificially ventilated infants and young children can be established by continuous intravenous infusion of midazolam.

Arya and Ramji [19] performed a double blind randomized placebo controlled trial to determine the efficacy of midazolam as a sedative in mechanically ventilated newborn infants for 7 days. Seventeen neonates with a body weight <2000 g received a bolus of midazolam of 200 *μ*g/kg followed by continuous infusion of 60 *μ*g/kg per hour. Sixteen infants received placebo. The midazolam group had significantly better sedation than the placebo group from 18 to 24 hours after enrollment. At 48 hours, there were no significant differences in sedation between midazolam and placebo groups. None of the infants had hypotension on loading midazolam. Sedation provided by continuous infusion of midazolam appeared to be comparable to morphine alone in newborn infants on mechanical ventilation. These authors observed that the behavioural assessment was used to demonstrate the sedative effect of midazolam in ventilated newborn infants. Opioid and benzodiazepines combination is most commonly used to sedate newborns, but their use is limited by prolonged variable elimination of these drugs, leading to prolonged sedation. Midazolam produces a rapid and consistent effect because of its short *t*_1/2_. Hypotension has been reported as an adverse effect of midazolam in few infants after intravenous administration of midazolam [28, 78]. Arya and Ramji [19] did not find hypotension in any neonates.

When minimal sedation is required, intranasal midazolam is an excellent sedative [79]. Intranasal strategies have been introduced to maximize the potential for successful sedation and to minimize risk. These authors reviewed the literature to evaluate the effectiveness of a second dose of intranasal midazolam. The strategy incorporates a second dose of intranasal midazolam, 10 to 15 min after the first dose, to obtain the desired level of anxiolysis. One hundred infants with an age ranging from 1 to 59 months were enrolled in the study. Eighty patients (80%) obtained satisfactory sedation effects. There were no reported complications, minimal untoward side reactions, and no delays in discharge. A second-dose of intranasal midazolam strategy was effective in achieving satisfactory minimal sedation in children. These authors stated that only a small proportion of patients would benefit from one dose of intranasal midazolam.

Intranasal midazolam use has been reported since 1988 [80]. The intranasal route is desirable because it obviates the need for intravenous access, avoids the pain of the intramuscular injection, and is easily accessible. Due to the reach vascular plexus of the nasal cavity and the communication to the subarachnoid space via the olfactory nerve and sheath, adequate cerebrospinal fluid levels can be achieved rapidly [81, 82]. The bioavailability of intranasal midazolam ranged from 50% to 83% [83–85]. The most common adverse effects reported following intranasal midazolam are burning or irritation in the nose and a bitter taste in the mouth [86–88].

Intranasal midazolam was studied by Lane and Schunk [89] using a unique atomization delivery system. Two hundred and five children admitted to the study, aged from 1.5 to 60 months, had mean ± SD age of 31.3 ± 13.2 months. The median initial dose of intranasal midazolam was 400 *μ*g/kg (range: 300 to 800 *μ*g/kg). Laceration repair was the most common procedure necessitating sedation (89%). Eleven patients (5.4%) required an additional sedative dose to complete the procedure. Ten of 11 patients received ketamine as the adjunctive sedative, and 1 patient required additional intranasal midazolam. There was 1 adverse event (0.5%). This was a minor desaturation episode following ketamine administration requiring a brief blow by oxygen. There were no adverse events in patients who received intranasal midazolam. These authors conclude that atomized intranasal midazolam is effective in providing anxiolysis to children undergoing minor procedures in the pediatric Department.

Harcke et al. [90] reviewed 162 consecutive sedations in which midazolam was employed. The patients' mean age was 4.1 years (range: 19 days to 19 years). Midazolam is effective in providing anxiolysis to children based upon a calculated dose of 200 to 400 *μ*g/kg. Intranasal midazolam has a rapid onset and is more convenient than intravenous agents, because of its short duration. A dose of 200 *μ*g/kg is safe and effective. In 142 of 162 cases (88%), midazolam demonstrated a positive effect; in 20 cases (12%), it was ineffective. The adverse effects were brief coughing, sneezing, or crying often occurring immediately after midazolam administration, but these were not considered adverse reactions unless they persisted. In 91 of 162 cases (56%), midazolam was used in conjunction with another agent. Fifteen adverse reactions (9%) occurred, including delay in awakening in 4 patients, reactive airway in 2 cases, desaturation and apnea in 4 cases, seizure in 1 case, agitation in 2 cases, and vomiting in 2 cases. Midazolam was the sole sedative in 71 of 162 (44%) cases. The use of midazolam to minimize the difficulty of starting an intravenous infusion constitutes the most common application. In 89% of the patients, it accomplished the desired effect. Of particular interest was the use of midazolam as a primary means of sedation since its intranasal route obviates the need for intravenous medication. An intranasally administered sedative with quick onset and short duration seems very innocuous.

### 3.10. Oral Administration of Midazolam in Neonates

Data describing midazolam disposition following oral administration to preterm infants are few. In literature, there are only two articles on the fate of midazolam following oral administration to neonates [2, 6]. A single oral bolus dose of 100 *μ*g/kg was administered to 15 preterm infants with gestational and postnatal ages ranging from 26 to 31 weeks and from 3 to 13 days, respectively. Mean ± SD of body weight was 1076 ± 240 g. In 8 out of these 15 patients, the pharmacokinetics of intravenous midazolam was also studied. Apparent oral Cl, apparent Vd, and plasma *t*_1/2_ are given as median and range. Cl was 2.7 mL/kg/min (range: 0.64 to 15.5 mL/kg/min), Vd was 1.4 L/kg (range: 0.3 to 12.1 L/kg), and *t*_1/2_ was 7.6 hours (range: 1.2 to 15.1) hours. The absolute bioavailability was 0.49 (range: 0.12 to 1.0).

In another study, De Wildt et al. [2] evaluated the pharmacodynamics and safety of midazolam after intravenous continuous infusion or oral administration. Patients were randomly assigned to initially receive an oral bolus dose of 100 *μ*g/kg midazolam. Pharmacodynamic measurements consisted of a COMFORT score at baseline and at 0.5, 1, 2, 4 and 6 hours after dose. A total of 24 infants were enrolled, of whom seven received both intravenous and oral midazolam, 13 infants received only intravenous midazolam, and 4 received only oral midazolam.

Overall, mean COMFORT score decreased (i.e., sedation increased) significantly within 30 min after intravenous administration and within 1 hour after oral administration (*P* = 0.003). No relationship was found between overall COMFORT scores or change in COMFORT score from baseline and midazolam. Diastolic blood pressure decreased significantly (approximately 11%) after intravenous, but not after oral, midazolam administration. No serious adverse events were reported. Midazolam administered as an intravenous infusion or oral bolus dose appears to be effective and well tolerated in a small majority of preterm infants. However, a considerable number of neonates did not respond to midazolam. The lack of response may be due to the fact that patients truly experienced therapeutic failure and/or consequent to the inability of the COMFORT score to adequately reflect sedation uniformly in sick preterm infants.

### 3.11. Management of Refractory Seizures in Neonates by Midazolam

Slaughter et al. [91] reviewed the literature on the pharmacological treatment of neonatal seizures. Their research was based on 557 initial articles and 14 additional studies after reference reviews, with 16 meeting inclusion criteria. Seizures suspected in high-risk neonates require confirmation of seizures with electroencephalogram. If seizures are confirmed by electroencephalogram, give phenobarbital 20 mg/kg intravenously and start phenobarbital maintenance 5 mg/kg per day. If seizures continue give additional 20 mg/kg intravenously. If seizures continue there are three possible drugs to take into consideration: levetiracetam (50 mg/kg intravenously, then 40 mg/kg per day maintenance, divided twice daily), phenytoin/fosphenytoin (20 mg/kg intravenously, and start a second maintenance medication, 5 mg/kg per day divided every 8 hours), or lidocaine (2 mg/kg intravenously bolus, then 6 mg/kg per hour, then titrate down by 2 mg/kg per hour, every 12 hours). If seizures continue, administer midazolam (0.15 mg/kg intravenous bolus, then 1 *μ*g/kg per min, up to a maximum of 18 *μ*g/kg per min). If seizures continue, consider phenobarbital drip or lidocaine drip if not yet tried (unless phenytoin/fosphenytoin has been used).

The neonatal period is one of the highest risk periods for seizures [92], which occurs in 1% to 5% of neonates [93] and is indicative of underlying neurologic dysfunction. Seizures are refractory to the first-line antiepileptic drugs, phenobarbital and phenytoin, in more than 50% of infants and children [94]. Midazolam succeeded to manage refractory neonatal seizures. In neonates, antiepileptic drugs can be even less effective in controlling seizures than in adults [95]. Little information is available on the use of midazolam in neonatal seizures. However, rapid control of status epilepticus with midazolam has been demonstrated with complete clinical and electrographic response in neonates who did not respond to phenobarbital and phenytoin [23, 24, 96].

Van Roij et al. [97] reviewed the treatment of neonatal seizures. Midazolam has also been used for phenobarbital refractory seizures with an efficacy between 67% and 80%. In other studies, midazolam was compared to lidocaine as a second-line anticonvulsive drug [24, 92, 98, 99]. In a study by Shany et al. [98], four of the eight neonates treated with midazolam showed a partial response. Three neonates with status epilepticus did not respond to phenobarbital and phenytoin [92] but they responded to midazolam infusion. Midazolam was infused at a rate of 0.10 to 0.13 *μ*g/kg per hour for a period of 43 to 54 hours. Midazolam may be considered a safe and effective antiepileptic drug in refractory neonatal seizures of diverse aetiologies.

Status epilepticus, a serious, life-threatening emergency, characterized by prolonged seizure activity, occurs most commonly in pediatric patients. Diazepam, phenytoin, or phenobarbital terminates seizures within 30 to 60 min, but there are patients who are refractory to standard therapy. Midazolam has emerged as a new treatment option. The review by Holmes and Riviello [100] compared the use of midazolam with that of phenobarbital in published reports of pediatric patients refractory status epilepticus. Most of status epilepticus can be controlled by benzodiazepines, phenytoin, or phenobarbital; in refractory cases that persisted beyond this therapy, the potential treatment options are less than ideal. The antiepileptic properties of midazolam were identified during the late 1970s, and more recently the drug has emerged as an effective treatment for resistant status epilepticus. Midazolam was well tolerated by pediatric patients with refractory status epilepticus. In the study by Holmes and Rivielo [100], only one incidence of mild hypotension was present in a 4-week-old infant. A number of published case reports showed the successful use of midazolam in terminating refractory status epilepticus in a total of 29 pediatric patients [100]. Although midazolam has not been directly compared with pentobarbital in a controlled study, the antiepileptic effectiveness and apparent safety of midazolam in children warrant its consideration as the initial treatment in pediatric cases of refractory status epilepticus [100].

Castro Conde et al. [24] stated that the outcome of 45 neonates with electroencephalogram-confirmed seizures was analyzed with regard to treatment. Seizures persisted in 17 of 32 neonates receiving phenobarbital/phenytoin. Thirteen neonates had a poor outcome and 4 died. In contrast, seizures were rapidly controlled in 13 of 13 nonresponders to phenobarbital/phenytoin treated with midazolam (0.15 mg/kg intravenous bolus, followed by continuous infusion of 1 *μ*g/kg per min, increasing by 0.5 to 1 *μ*g/kg per min every 2 min until a favourable response or a maximum of 18 *μ*g/kg per min). If the status epilepticus persisted after the initial bolus, another bolus of 100 to 150 *μ*g/kg was administered 15 to 30 min later. Nonresponders to phenobarbital/phenytoin were controlled by midazolam.

The efficacy of a combination of midazolam and phenytoin in treating generalized convulsive status epilepticus was studied in 122 children with the median age of 24.4 months (range: 0.5 to 197 months) [101]. Forty-three percent of patients required artificial ventilation. Thirty-two percent of patients developed respiratory insufficiency during initial therapy with midazolam. Three patients had an apnea, and 8 patients were intubated after a bolus of midazolam. Twenty-two percent of patients were artificially ventilated to protect the airway. No deaths were attributable to generalized convulsive status epilepticus itself. The treatment protocol consisted of a stepwise use of midazolam and phenytoin. *Level 1*: rectal midazolam of 500 *μ*g/kg, or 100 *μ*g/kg, intravenously was administered. *Level 2*: after 10 min, 20 mg/kg phenytoin was administered intravenously. *Level 3*: after phenytoin load, 200 *μ*g/kg midazolam was administered intravenously as a bolus, followed by a continuous infusion of 100 *μ*g/kg per hour of midazolam, increased by 100 *μ*g/kg per hour every 10 min, after extra loading of 100 *μ*g/kg, to a maximum of 1 mg/kg per hour. *Level 4*: phenobarbital 20 mg/kg intravenously, or pentobarbital 2 to 5 mg/kg intravenous load, 1 to 2 mg/kg per hour of phenobarbital was continuously intravenously infused. Eighty-two of 122 infants (68%) received initial rectal diazepam. Cessation of epileptic activity was achieved with midazolam in 58 patients (48%). Midazolam is an effective agent for seizure control [102]. Several studies in a small number of children have described the effective use of intravenous, intramuscular, rectal, nasal or sublingual midazolam [96, 102–104].

Hu et al. [23] determined the efficacy and safety of continuous midazolam infusion in neonates with uncontrollable neonatal seizures. Thirty-two neonates whose seizures could not be controlled by diazepam, phenytoins or phenobarbital were enrolled in the study. Midazolam was given as an intravenous bolus dose followed by a continuous infusion. The maximum infusion of midazolam ranged from 1 to 15 *μ*g/kg per min, with an average of 4 *μ*g/kg per min. There were no significant changes in serum sodium, potassium, calcium, or glucose in any of the patients. Four patients had recurrent seizures, which stopped after midazolam was reinstituted. Adverse effects included hypotension in 12 patients (38%), that was successfully controlled with dopamine and/or dobutamine, and transient urinary retention in 38% of infants. All the patients enrolled in the study had a successful management of seizures. These results suggest that midazolam is safe and effective for the treatment of uncontrollable seizures.

Midazolam is a highly effective antiepileptic agent, even in patients who have not responded to other benzodiazepines [102]. Compared with phenobarbital, midazolam has fewer hemodynamic consequences, minimizing the need for invasive monitoring. The need for endotracheal intubation and mechanical ventilation also appears to be less frequently necessary with midazolam. Patients recovery after midazolam is shorter than with phenobarbital. Although midazolam has not been directly compared with phenobarbital in a controlled study, the antiepileptic effectiveness and apparent safety of midazolam in children warrant its consideration as the initial treatment in pediatric cases of refractory status epilepticus.

Sheth et al. [96] studied six neonates with gestational and postnatal ages ranging from 30 to 41 weeks and 1 to 9 days, respectively. These neonates developed seizures from varying causes. In each case, seizures persisted for >12 hours despite high dose of phenobarbital with or without the addition of phenytoin. All infants received intravenously 20 to 40 mg/kg of phenobarbital. Maintenance phenobarbital dosage was approximately 8 mg/kg and was administered in two divided doses. Four of the six infants also received 20 mg/kg of phenytoin. Three infants were suffering from focal clonic seizures; two infants had tonic seizures and one had myoclonic seizures. Onset of the seizure was between 1 and 24 hours except in the patients with meningitis, in whom the onset was at 9 days. All infants received a standard intravenous loading dose of 20 mg/kg and subsequently an additional 20 to 40 mg/kg of phenobarbital. Maintenance phenobarbital dosage approximated 8 mg/kg and was administered in two divided doses. Four of the six infants also received 20 mg/kg of phenytoin. In all six infants, midazolam infusion was started with a loading dose of 150 *μ*g/kg and then maintained between 100 and 400 *μ*g/kg per hour. After the loading dose, clinical seizures were no longer observed in four infants. The remaining became seizure free within one hour after the administration of midazolam. These results show that midazolam administrated by continuous intravenous infusion may be a valuable adjunct in the management of refractory neonatal seizures.

### 3.12. The Safety and Efficiency of Midazolam versus Diazepam for the Treatment of Febrile Seizures in Children

McIntyre et al. [105] compared rectal diazepam and buccal midazolam for emergency room treatment in 177 children, who had a median age of 3 years, with acute febrile seizures. The dose of diazepam and midazolam ranged from 2.5 to 10 mg. The primary endpoint was the cessation of seizures within 10 min and for at least 1 hour, without respiratory depression requiring ventilation. The therapeutic success was 56% for buccal midazolam and 27% for rectal diazepam. The rate of respiratory depression did not differ between groups. Buccal midazolam was more effective than rectal diazepam for children presenting to hospital with acute febrile seizures and was not associated with increased incidence of repertory depression.

Mahmoudian and Zadeh [106] determined whether intranasal midazolam is as safe and effective as intravenous diazepam in the treatment of acute childhood seizures. Seventy children aged between 2 months and 15 years with acute febrile seizures were admitted to the “pediatric emergency department” of a general hospital. Intranasal midazolam (0.2 mg/kg) and intravenous diazepam (0.2 mg/kg) were administered after intravenous lines were established. Intranasal midazolam and intravenous diazepam were equally active. The main time to control seizures was 3.58 ± 1.68 min (midazolam) and 2.94 ± 2.62 (diazepam). No significant side effects were observed in either groups. Although intranasal midazolam was as safe and effective as diazepam, seizures were controlled more quickly with intravenous diazepam than with intranasal midazolam.

### 3.13. Comparison of the Sedatives Effects of Midazolam with Those of Other Sedatives in Neonates

Many agents can depress the function of the central nervous system producing calming or drowsiness (sedation). Sedation may be performed with opioids (fentanyl, morphine, and diamorphine), with the sedative-hypnotic midazolam and with the aesthetic propofol in ventilated neonates.

Endotracheal intubation of premature infants is performed frequently in the “neonatal intensive care units” and delivery room. The procedure is associated with physiological and biochemical responses, and now there is strong evidence that premedication (sedation and analgesia) improves the physiological stability, decreases the difficulty of procedure, and also reduces the potential for airway injury [107].

Penido et al. [108] compared intubation conditions between midazolam and propofol in a blinded, randomized, and controlled study in 20 neonates. Ten patients, with a gestational age of 32 ± 1.6 weeks and with a bodyweight of 1653 ± 357 g, were treated with midazolam as an intravenous bolus of 200 *μ*g/kg and 1 *μ*g/kg remifentanil. The remaining 10 patients, with a gestational age of 31.5 ± 1.5 weeks and a body weight of 1523 ± 35 g, were treated with 2,000 *μ*g/kg propofol and 1 *μ*g/kg remifentanil. All neonates were in nasal continuous airway pressure therapy before intubation, with similar ventilatory parameters and degree of respiratory system. No statistical differences were observed as well between the drugs regarding pain and stress level before and after the intubation using the NIPS scale. No differences were observed in heart rate and blood pressure between the two drugs. Adverse effects such as hypotension were observed in two infants in each drug, and bradycardia was seen in one infant in the propofol group. The present findings did not show any difference in the quality of intubation, presence of adverse effects, changes on the hemodynamic variables, and quality of sedation and analgesia achieved when midazolam or propofol was used as a hypnotic associated with remifentanil as premedication for tracheal intubation in preterm neonates. The pharmacokinetics of propofol was studied in 25 neonates after a bolus injection of 3000 *μ*g/kg [109] and found that there is a remarkable variation in the propofol Cl which may be influenced by postmenstrual and postnatal ages.

Malagon et al. [25] compared the effect of three anaesthetics: midazolam, propofol, and sevoflurane, on the postoperative production of cardiac troponin T in 90 pediatric patients undergoing cardiac surgery. Patients received pre-medication consisting of oral atropine (20 *μ*g/kg) and midazolam (500 *μ*g/kg) 30 min before induction of anaesthesia. Anaesthesia was induced with sevoflurane (1 *μ*g/kg), followed by a bolus of sufentanil (1 *μ*g/kg) and pancuronium (200 *μ*g/kg). The groups were comparable with respect to sex, age, weight, type of surgery, cardiopulmonary bypass, aortic cross clamp, and circulatory arrest time. The values for acyanotic patients were similar with midazolam, propofol, and sevoflurane. The corresponding values for cyanotic patients were similar to the three anaesthetics. There was a significant correlation between ventilator hours and troponin T concentrations, at the time of 8 hours, in the sevoflurane group (*r* = 0.45) and midazolam group (*r* = 0.50) but not in the propofol group. The present findings show that the postoperative production of troponin T, in pediatric patients undergoing cardiac surgery, is similar to midazolam, propofol, or sevoflurane anaesthesia.

Hartwig et al. [17] studied the continuous intravenous sedation using midazolam and fentanyl in patients recovered in the pediatric intensive care unit. Twenty-four artificially ventilated children, aged from 26 days to 5 years (17 infants younger than 1 year) received an intravenous midazolam infusion of 100 *μ*g/kg per hour. Fifteen of the 24 children received a combination of midazolam with fentanyl; the latter was infused at a rate of 0.5 to 2 *μ*g/kg per hour. The mean duration of fentanyl infusion was 64 hours (range: 20 to 188 hours). No cardiovascular or respiratory adverse effects were seen. After stopping the fentanyl infusion, the children were sedated with midazolam. With midazolam as the only sedative drug, a significantly (*P* < 0.05) higher mean infusion rate of midazolam was necessary to reach a reliable sedation and it was of 209 *μ*g/kg per hour (range: 100 to 500 *μ*g/kg per hour). Mean serum concentration of midazolam decreased from 513 *μ*g/L (range: 124 to 1,093 *μ*g/L) to 330 *μ*g/L (range: 58 *μ*g/L to 1,240 *μ*g/L) after 4 to 5 days. Midazolam Cl increased in relation to the duration of midazolam therapy (*r* = 0.544; *P* < 0.01). In the early phase of midazolam therapy, Cl of midazolam was 5.8 + 3.8 mL/kg per min and increased significantly by 133% to 13.6 + 10.6 mL/min per min in the late phase of therapy. The reason for the increase of midazolam Cl may be either an increase of the Vd or an increase of the hepatic Cl due to an enzyme induction of midazolam or by increasing hepatic blood flow during a treatment over a longer period. During the first 24 to 72 hours of midazolam therapy, the sedation score was significantly correlated with the serum midazolam concentration (*r* = 0.76; *P* < 0.001). The desirable sedation score could be achieved during this time period at a serum concentration between 100 and 500 ng/mL with an infusion rate of 100 to 400 *μ*g/kg per hour. Midazolam seems to guarantee reliable sedation and anxiolytic action. The evaluation of the sedation score showed that reliable sedation can be achieved at a midazolam infusion of 100 to 400 *μ*g/kg per hour and at a serum midazolam concentration between 100 and 500 ng/mL. In combination with fentanyl, the midazolam infusion rate could be held at a lower level. Continuous intravenous infusion of fentanyl and midazolam has been shown to provide satisfactory analgesia.

Ketamine and midazolam are commonly used in children undergoing cardiac catheterization. Jobeir et al. [110] reviewed pediatric cardiac catetherization procedures in 154 patients (age from 0.3 to 192 months) who underwent a total of 205 procedures. They received ketamine (*n* = 79; 1.05 ± 0.88 mg/kg per hour), midazolam (*n* = 35; 1.57 ± 1.03 mg/kg per hour), or both (*n* = 91; ketamine, 1.13 ± 0.84 mg/kg per hour, and midazolam, 1.57 ± 1.03 mg/kg per hour). In 18.5% of patients there were complex cardiac lesions. Pre- and postprocedure systolic and diastolic mean blood pressure were 72 ± 14 and 68 ± 12 mmHg, respectively. Mean procedure time was 79 ± 36.2 min. The anaesthesiologist's assistance was required by the cardiologist in 21 procedures. Pre- and postprocedure O_2_ saturation were 93.19 ± 8.72 and 93.63 ± 8.3, respectively. The mortality was zero. The two groups were not different in relation to the drug use (*P* = 0.283) or the complexity of the cardiac lesion (*P* = 0.051). However, there was significant difference between the two regarding the need for supporting drugs (3/21 versus 3/184; *P* = 0.02) or oxygen treatment (7/21 versus 26/184; *P* = 0.014). These authors conclude that low dose of ketamine and midazolam can be administered safely to most pediatric patients by the cardiologist, who can safely predict the need for an anaesthesiologist.

### 3.14. Adverse Effects of Midazolam in Neonates

Midazolam is incompatible with fat emulsion, albumin, ampicillin, bumetanide, cefepime, ceftazidime, dexamethasone, fosphenytoin, furosemide, hydrocortisone succinate, micafungin, nafcillin, dimenhydrinate, perfenazine, ranitidine hydrochloride, thiopental, prochlorperazine edisylate, and sodium bicarbonate [3, 111]. Ketoconazole and erythromycin are potent inhibitors of CYP3A4 and must not be used in association with midazolam.

There have been reports of life-threatening adverse respiratory and cardiovascular events occurring after administration of midazolam. Pain, tenderness, and thrombophlebitis have occurred following injection of midazolam. Hiccups have been reported. Death due to respiratory depression, hypotension, or cardiac arrest has been reported in patients given intravenous midazolam for conscious sedation [111].

Midazolam is not free from adverse effects when administered to neonates. The first intravenous loading dose of midazolam administered to premature infants not infrequently causes respiratory depression, with hypotension, a fall in cerebral blood flow, and paradoxical agitation [1]. The paradoxical effect such as hyperexcitability and myoclonus may be responsible for the low number of GABA_A_ receptors in the neonate [72].

Respiratory depression and hypotension are common when midazolam is used in association with narcotics or following rapid bolus administration. Hypotension has also been reported after continuous infusion of midazolam. The percentage of infants who develop hypotension varies with the studies. Seizure-like myoclonus has been reported in 8% of premature infants receiving continuous infusion [3]. This also may occur following rapid bolus administration and in patients with underlying disorders of the central nervous system. Nasal administration may be uncomfortable because of a burning sensation [3]. Drug accumulation may occur with repeated doses, prolonged infusion therapy of midazolam, or concurrent administration of cimetidine, erythromycin, or fluconazole [3].

Van den Anker and Sauer [112] observed several adverse effects in premature neonates with a gestational age below 32 weeks. Heart rate and arterial blood pressure decreased directly following an intravenous bolus injection of 200 *μ*g/kg midazolam, but the most disturbing observation was the appearance of involuntary epileptiform movements lasting for 15 to 30 sec. The pathogenesis of this phenomenon is speculative, but perhaps in these preterm infants the decrease in arterial pressure and heart rate has also an impact on cerebral blood flow or there is a direct effect of midazolam on the brain.

Ng et al. [21] recently reviewed the effects of midazolam administered intravenously to infants aged 28 days or less for sedation. These authors reached the conclusion that there are no sufficient data to administrate midazolam intravenously for sedation to neonates undergoing intensive care. Since brain maturation is incomplete in infants, side effects of centrally acting drugs may differ from those in older patients [75].

The major adverse effects associated with continuous infusion of midazolam are related to tolerance and an associated abstinence or withdrawal syndrome [71, 76, 113, 114]. Withdrawal syndromes with central nervous system manifestations have been observed. These syndromes have been characterized by seizures, agitation, inability to communicate, and hallucinations or by somatic manifestations such as vomiting, tachycardia, and fever. Bergman et al. [114] suggested that prolonged administration of midazolam to infants, especially to those who had hypoalbuminemia or who were receiving concomitant fentanyl, may produce a long-lasting encephalopathy. Hepatic biotransformation do not mature until 5 months of age; midazolam is metabolized slowly in young infants. Since brain maturation is incomplete in this age group, side effects of centrally acting drugs, including manifestations of an abstinence syndrome, may differ from those in older patients. Midazolam is highly bound to plasma protein (98% in adults [5]); low serum albumin concentrations may result in higher brain levels and may exacerbate the effects of midazolam by increasing the plasma concentration of unbound drug [115].

Harte et al. [116] evaluated the effects of intravenous midazolam injection on cerebral blood flow in very low birthweight ventilated infants. Ten infants, with birthweight ≤1500 g, were treated with an intravenous bolus of 100 *μ*g/kg midazolam. No change in heart rate occurred during the study period, while mean arterial blood pressure decreased by 3 mmHg, 5 min after midazolam administration, compared to baseline values. A nonspecific fall in transcutaneous PCO_2_ was seen at 20 min after midazolam administration. Mean cerebral blood flow decreased from baseline by 12% at 5 min after midazolam administration then returned to predose values. Midazolam concentrations in the therapeutic range were proved to be effective in sedation of pediatric intensive care infants. As only minor cerebral and hemodynamic effects were found with the use of midazolam in stable ventilated preterm infants, it appears that midazolam is a safe, short-term sedative agent.

The administration of a loading dose of 200 *μ*g/kg midazolam, followed by a constant infusion of 200 *μ*g/kg per hour, to 11 ventilated premature infants, caused changes in cerebral oxygenation and hemodynamics [117]. Van Leuven et al. [118] reported a brief and moderate suppression of the electroencephalogram background pattern, lasting less than 2 hours in 4 out of 15 infants, after a loading dose of 50 *μ*g/kg midazolam, followed by a continuous infusion of 150 *μ*g/kg per hour. Harte et al. [116] reported minor cerebral and hemodynamic effect after the intravenous bolus administration of 100 *μ*g/kg of midazolam to 10 infants with a birthweight ≤1500 g.

## 4. Discussion

The present paper reviews the clinical pharmacology of midazolam; most of the work was performed in neonates, and several data have been obtained in children. Benzodiazepines provide effective control of patient agitation without imposing a significant load on the cardiovascular system. Midazolam is a short-acting benzodiazepine with rapid onset of sedation and is preferable to diazepam. *t*_1/2_ of midazolam is 22-fold shorter than that of diazepam [5]. In adults, midazolam *t*_1/2_, Cl, and Vd are 1.9 hours, 6.6 mL/kg per min, and 1.1 L/kg, respectively. In healthy preterm infants, *t*_1/2_ is 3.3-fold longer, Cl is 4.4-fold smaller, and Vd is 1.1 L/kg in adult and neonates. The rate of midazolam elimination is slower in infants than in adults. Midazolam is mainly eliminated by metabolism in adults. It is hydroxylated in position 1 to form 1-hydroxymidazolam by CYP3A4 and CYP3A5. These enzymes surge in the liver during the first weeks of life [8, 9, 11] and thus their activities and, consequently, the metabolic rate of midazolam are lower in neonates than in older infants and adults. These findings explain the longer *t*_1/2_ and the slower Cl observed in neonates than in older infants and adults.

Children, with an age greater than 2 years, had a Cl of midazolam that ranged from 5.8 to 13.6 mL/kg per min (mean ± SD = 9.6 ± 3.5 mL/kg/min) [17, 47]. The work based on 18 patients, aged from 2 days to 17 years, with half of the patients having an age less than 6 months, revealed that Cl of midazolam was 5.0 ± 3.9 mL/kg per min [34]. This estimate is lower than that reported in patients with age greater than 2 years [17, 47]. Thus, Cl of midazolam increases with the age up to at least 2 years of life.

Disease affects the pharmacokinetics of midazolam. A patient suffering from renal failure, with malignant hypertension, and having a creatinine concentration of 691 *μ*mol/L and treated with midazolam, had a *t*_1/2_, Vd, and Cl of this drug 6.3-fold longer, 14.5-fold greater, and 2.6-fold larger, respectively, [34] than the normal values [26]. Multiple organ failure reduces the Cl of midazolam [27]. The severity of disease lowers the Cl of midazolam, likely by reducing the activities of CYP3A4 and CYP3A5.

Fifteen preterm infants, requiring mechanical ventilation, were treated with midazolam continuous infusion of 60 *μ*g/kg per hour [28]. They had a *t*_1/2_ 1.9-fold longer than the normal value [26]. Some of these infants were treated with different drugs; 7 infants received fentanyl, a substrate of CYP3A4 [52]. Fentanyl may compete with the metabolism of midazolam reducing the midazolam hydroxylation rate, thus lengthening the midazolam *t*_1/2_. The long *t*_1/2_ of midazolam observed by Jacqz-Aigrain et al. [28] may reflect the effect of the mechanical ventilation and the interaction with drugs coadministered with midazolam. ECMO therapy increases several times *t*_1/2_, Cl, and Vd of midazolam [31, 119].

In literature, there are three studies on the population pharmacokinetics of midazolam [32–34]. Burtin et al. [32] studied 187 neonates, with a gestational age ranging from 26 to 42 weeks, and Lee et al. [33] studied 60 preterm infants, with a gestational age ranging between 24 and 31 weeks. All these infants underwent mechanical ventilation. Mean *t*_1/2_ was 1.6-fold greater [32] than the normal value [26] and 2.2-fold greater [33] than normal estimate [26]. These findings suggest that mechanical ventilation may increase midazolam *t*_1/2_ in neonates. The infants studied by Lee et al. [33] had a smaller gestational age than the infants studied by Burtin et al. [32]. The longer *t*_1/2_ observed by Lee et al. [33] might, at least in part, reflect the different gestational ages. The population kinetics of midazolam was studied in 18 pediatric patients, aged from 2 days to 17 years; half of the patients had an age < 6 months [34]. Midazolam *t*_1/2_, Cl, and Vd were 5.5 ± 3.5 hours, 5.0 ± 3.9 mL/kg per min, and 1.7 ± 1.1 L/kg, respectively. These parameters were 0.87-fold lower, 3.0-fold higher, and 1.5-fold larger than the normal values. These data suggest that the Cl of midazolam increases with the neonatal development.

The data on midazolam pharmacodynamics in neonates are few and do not allow any conclusion. De Wildt et al. [54] did not find any relationship between sedation level category and drug concentrations. Vet et al. [27] did not find a correlation between inflammation severity and COMFORT score. Trouiller et al. [64] and Arbour et al. [65] stated that the optimal measure, to monitor the pharmacodynamic endpoint, still needs to be determined.

Neonates may undergo uncomfortable procedures and may experience moderate to severe pain in the “neonatal intensive care unit.” The availability of an appropriate sedative that reduces the stress and pain is desirable. Midazolam is an effective sedative and represents a convenient choice among the sedatives because of its fast onset of action and the rapid termination of effect. The use of midazolam for newborn sedation remains empirical, and further research on the effectiveness and safety of midazolam in ventilated neonates is needed before its employment can be considered in routine clinical use [22, 111].

Sedation of neonates with midazolam may be performed by intravenous administration (bolus or continuous infusion) or by intranasal administration when minimal sedation is required [79]. The median initial dose of intranasal midazolam is 400 *μ*g/kg (range: 300 to 800 *μ*g/kg). The bioavailability of intranasal midazolam ranged from 50% to 83% [83–85]. A second dose of intranasal midazolam, 10 to 15 min after the first dose, to obtain the desired level of sedation, may be administered. The oral administration of midazolam is not used in neonates and only two reports were found [2, 6]. No information is available in literature on the sublingual administration of midazolam to neonates.

Treluyer et al. [77] determined the minimal effective dose of intravenous midazolam required for appropriate sedation in 95% of infants, 1 hour after sedation. The sedation procedure was considered a success if all the following criteria were met: no agitation, no grimacing, and no crying facial expression before and during tracheal suctioning. Administering 200 *μ*g/kg midazolam, the final estimated probability of success is 76.9%. Using lower midazolam concentrations, the probability of success decreased.

Mulla et al. [30] administered diazepam to 45 infants to obtain a plasma midazolam concentration between 200 and 1000 ng/mL within 24 hours to start treatment. Infants, with age greater than 33 weeks of gestation, received a continuous infusion of midazolam of 60 *μ*g/kg per hour. Infants, below 33 weeks of gestation, received the same continuous infusion rate during the first 24 hours of treatment, followed by a continuous infusion of 30 *μ*g/kg per hour of midazolam. Mean midazolam concentrations were 634 ± 234 ng/mL, 628 ± 327 ng/mL, and 543 ± 327 ng/mL at 24 and 48 hours and at the end of treatment. Remarkable variability in midazolam plasma concentrations was observed, but none of the infants had levels of midazolam below 200 ng/mL and only 2 infants had a plasma concentration of midazolam above 1000 ng/mL.

A dose of midazolam between 50 and 400 *μ*g/kg per hour produced midazolam concentrations sufficient for sedation of infants [17]. Dosage of midazolam must be increased during therapy due an increase of midazolam Cl. Arya and Ramji [19] administered midazolam as a bolus of 200 *μ*g/kg, followed by a continuous infusion of 60 *μ*g/kg per hour, to 17 infants with a body weight <2000 g, undergoing mechanical ventilation. No significant adverse effects were observed. The course of mechanical ventilation was not influenced by the use of midazolam. These authors observed that the behavioural assessment was used to demonstrate the sedative effect of midazolam in ventilated infants.

Data describing midazolam pharmacokinetics following oral administration to neonates are few; only two articles are available in literature [2, 6]. A single oral dose of 100 *μ*g/kg was administered to infants (*n* = 15) with gestational age ranging from 26 to 31 weeks. The apparent oral Cl and Vd and the plasma *t*_1/2_ (given as median) were 2.7 mL/kg per min, 1.4 L/kg, and *t*_1/2_ 7.6 hours, respectively. These values are not considerably different from the normal values. The absolute bioavailability “F” of midazolam was 0.49.

The neonatal period is one of the highest risk periods for seizures [92], which occur in 1% to 5% of neonates [93]. Little information is available on the treatment of neonatal seizures by midazolam. This drug emerged as an antiepileptic drug in the 1970s, and more recently it has become an effective agent for the treatment of status epilepticus refractory to standard antiepileptic treatment. Rapid control of the status epilepticus has been demonstrated with midazolam. This drug produced a complete clinical and electrographic response in neonates refractory to phenobarbital and phenytoin [23, 24, 96].

The efficacy of a combination of midazolam and phenytoin, in treating generalized convulsive status epilepticus, was studied in 122 children with the median age of 24.4 months [101]. Forty-three percent of infants required artificial ventilation. Thirty-two percent of the patients developed respiratory insufficiency during initial therapy with midazolam. Eight-nine percent of infants managed on midazolam and phenytoin.

Midazolam successfully managed seizures in 32 neonates who did not respond to diazepam, phenytoin, or phenobarbital [23]. Midazolam was given as an intravenous bolus dose of 100 *μ*g/kg followed by a continuous infusion of 1 to 15 *μ*g/kg per min. Twelve patients (38%) developed hypotension. Midazolam emerged as a treatment option against the status epilepticus in patients who did not respond to diazepam, phenytoin, or phenobarbital [100]. Compared with phenobarbital, midazolam has fewer hemodynamic consequences, minimizing the need for invasive monitoring [102].

Many drugs depress the function of the central nervous system producing sedation in ventilated infants. They are the opioids (fentanyl, morphine, and diamorphine), the sedative-hypnotic midazolam, and the anaesthetic propofol. Mahmoudian and Zadeh [106] did not find statistical differences regarding pain, stress level, and adverse effects between midazolam and propofol.

Hartwig et al. [17] experienced the treatment of midazolam with that of fentanyl in patients recovered in the “neonatal intensive care unit”. Twenty-four children were artificially ventilated for respiratory support after cardiac surgery. Their age ranged from 26 days to 5 years (17 infants were younger than 1 year) and they received an intravenous bolus of 100 or 200 *μ*g/kg midazolam followed by an infusion of this drug at a rate of 100 *μ*g/kg per hour. Fifteen of the 24 infants received a combination of midazolam with fentanyl. The latter was infused at a rate of 0.5 to 2 *μ*g/kg per hour. No cardiovascular or respiratory adverse effects were seen. Midazolam guarantees reliable sedation and anxiolytic action. With midazolam, as the only sedative drug, a significantly higher mean infusion rate of 209 *μ*g/kg per hour (range: 100 to 500 *μ*g/kg per hour) was necessary to reach a reliable sedation. In the early phase of midazolam therapy, Cl was 5.8 ± 3.8 mL/kg per min and increased by 133% to 13.6 ± 10.6 mL/kg per min in the late phase of therapy.

Ketamine was infused at a rate of 1050 ± 880 *μ*g/kg per hour (*n* = 79) and midazolam infusion was 140 ± 90 *μ*g/kg per hour (*n* = 35). There were no differences between the two groups regarding the need for supporting drugs or oxygen treatment. In 18.5% of patients there were complex cardiac lesions. No difference was found in relation to the drug used (*P* = 0.283) or the complexity of cardiac lesions (*P* = 0.051). These authors conclude that low dose of ketamine and midazolam can be administered safely to most pediatric patients by the cardiologist, who can safely predict the need for an anaesthesiologist.

Life-threatening adverse respiratory and cardiovascular events have been reported in adults after midazolam administration. Pain, tenderness, and thrombophlebitis have occurred following intravenous midazolam administration [109]. In neonates, intravenous midazolam may cause respiratory depression, with hypotension and a fall in cerebral blood flow. Myoclonus is sometimes seen, and paradoxical agitation has been reported [1]. The paradoxical effect such as hyperexcitability and myoclonus are due to the low number of GABA_A_ receptors present in the neonate [72].

The major adverse effects associated with continuous infusion of midazolam are related to tolerance and an associated abstinence or withdrawal syndrome [21, 71, 76, 112]. One hundred *μ*g/kg midazolam reduces the mean arterial blood pressure by 12% at 5 min after midazolam [114]. Hypotension may occur after continuous intravenous infusion of midazolam to neonates. The percentage of neonates who developed hypotension varies with the studies. Hu et al. [23] observed hypotension in 38% of infants treated with a continuous infusion of midazolam, ranging from 1 to 15 *μ*g/kg per min, to treat seizures. Twenty-seven percent of the infants receiving a continuous midazolam infusion of 60 *μ*g/kg per hour developed hypotension [28]. These infants were treated with several drugs and the contribution of these drugs in developing hypotension is unknown. Hypotension has been reported in few infants, with respiratory distress syndrome, who received a midazolam infusion of 60 *μ*g/kg per hour [78].

In conclusion, midazolam is a safe and effective drug, which may be used as a sedative or as an antiepileptic for the treatment of infants who are refractory to standard antiepileptic therapy. Midazolam is particularly useful in the treatment of the status epilepticus in infants. Midazolam is also used as an anaesthetic, but information about midazolam as an anaesthetic is lacking in neonates. Midazolam is extensively hydroxylated by two cytochrome P-450 forms, namely, CYP3A4 and CYP3A5. These enzymes surge in the liver during the first weeks of life. Neonates have a lower expression of these enzymes than older infants and adults. Consequently, the metabolic rate of midazolam is lower in neonates. Diseases affect the pharmacokinetics of midazolam; they may reduce the Cl or prolong *t*_1/2_ of midazolam. It is likely that these effects are due to the lowering of CYP3A4 and CYP3A5 activities. We feel that further research is required to ensure that the doses of midazolam recommended for the treatment of neonates are evidence-based.

## Figures and Tables

**Table 1:** Demographic data of patients and pharmacokinetic parameters of midazolam in neonates and children.

Comments	GA (weeks)	PNA (days)	BW (g)	Dose of midazolam	Number of infants	*t*_1/2_ (hours)	Cl (mL/kg/min)	Vd (L/kg)	Reference
Healthy neonates									
Intravenous administration	26 to 34	3 to 11	na	100 µg/kg Bolus IV	24	6.3^a^	1.8^a^	1.1^a^	[26]

Critically ill neonates and children									
Oral administration, the values are corrected for *F* = 49%	26 to 31	3 to 13	1,076 ± 240	100 µg/kg Bolus PO	15	7.6^b^	2.7^b^	1.4^b^	[6]
Note A	2 days 17 years	—	3.8 to 24.5 (kg)	90 µg/kg/h Infusion	21	na	0.14^a^ L/kg/h (diseased) 0.28^a^ L/kg/h (healthy) *P* = 0.0350	na	[27]
Infants required mechanical ventilation. Some drugs were administered	33 ± 3.3	1 to 5	1,900 ± 700	Infusion 60 µg/kg/h	15	12 ± 0.6	1.7 ± 1.8	1.2 ± 0.6	[28]
Respiratory distress syndrome (*n* = 5) and neonatal infection (*n* = 5)	37 ± 2.3	2 to 5	3,150 ± 520	200 µg/kg Bolus IV	10	6.5 ± 2.3	2.0 ± 1.2	0.9 ± 0.3	[29]

Neonates undergoing “extracorporeal membrane oxygenation” (ECMO)									
Note B	39.5 ± 1.9	3.8^a^	3,400 ± 600	50 to 250 µg/kg/h Infusion	10	6.8^a^ Initial	1.4^a^ Initial	3.1^a^ (L) Initial	[30]
	39.5 ± 1.9	3.8^a^	3,400 ± 600	50 to 250 µg/kg/h Infusion	10	33.3^a^ Terminal	4.1 Terminal	14.2^a^ (L) Terminal	
Median ECMO duration was 70 to 275 hours	40.4^a^	0.79^a^	3,000^a^	200 µg/kg Bolus IV	20	1.8^a^ Initial	2.6^a^ Initial	1.4^a^ Initial	[31]
*t*_1/2_ from the onset to steady-state was prolonged 5-fold	40.4^a^	0.79^a^	3,000^a^	200 µg/kg Bolus IV	20	na	7.6^a^ Terminal	4.9^a^ Terminal	

Population pharmacokinetics of midazolam in neonates and children									
Note D	26 to 42	0 to 9	700 to 5,200	Note C	187	9.9^a^	1.2 ± 0.2	1.0 ± 0.2	[32]
Note E	24 to 31	2 to 15	523 to 1,400	100 µg/kg Bolus IV	60	14.1^a^	0.938^a^	1.15^a^	[33]
Age ranged from 2 days to 17 years	—	—	3.5 to 60	50 to 400 µg/kg/h Infusion	18	5.5 ± 3.5	5.0 ± 3.9	1.7 ± 1.1	[34]

Adult subjects									
Adults	—	—	—	—	—	1.9 ± 0.6	6.6 ± 1.8	1.1 ± 0.6	[5]

Figures are the mean ± SD unless otherwise stated. In some cases, results are given as a range. *F*: oral bioavailability which is 49% [6]; ^a^mean, standard deviation is not available; ^b^median; GA: gestational age; PNA: postnatal age; BW: body weight; *t*_1/2_: *β*-phase elimination; Cl: clearance; Vd: apparent volume of distribution; na: not available; IV: intravenously; PO: by mouth; Note A: neonates were suffering from congenital heart disease (*n* = 4), upper airway infection (*n* = 4), pneumonia (*n* = 2), postcardiac surgery (*n* = 2), pulmonary hypertension (*n* = 2), and other (*n* = 7); Note B: midazolam was administered via a central or peripheral venous catheter; Note C: midazolam was administered intravenously as a continuous infusion to 109 infants, as a bolus dose to 22 infants, and as a combination of both to 56 infants. The bolus dose was 210 ± 239 µg/kg. The continuous infusion was 69 ± 63 µg/kg per h; Note D: all neonates received midazolam during artificial ventilation; Note E: neonates undergoing mechanical ventilation had a body weight <1,500 g.
